# Regularized Optimal Transport Based on an Adaptive Adjustment Method for Selecting the Scaling Parameters of Unscented Kalman Filters

**DOI:** 10.3390/s22031257

**Published:** 2022-02-07

**Authors:** Chang Ho Kang, Sun Young Kim

**Affiliations:** 1 Department of Mechanical System Engineering, Kumoh National Institute of Technology, Gumi 39177, Korea; kcguri@kumoh.ac.kr; 2 Department of Aeronautics, Mechanical and Electronic Convergence Engineering, Kumoh National Institute of Technology, Gumi 39177, Korea; 3 School of Mechanical Convergence System Engineering, Kunsan National University, Gunsan 54150, Korea

**Keywords:** regularized optimal transport (ROT), scaling parameter adaptation, Sinkhorn–Knopp algorithm, unscented Kalman filter (UKF), stability analysis on UKF

## Abstract

In this paper, an adaptation method for adjusting the scaling parameters of an unscented Kalman filter (UKF) is proposed to improve the estimation performance of the filter in dynamic conditions. The proposed adaptation method is based on a sequential algorithm that selects the scaling parameter using the user-defined distribution of discrete sets to more effectively deal with the changing measurement distribution over time and avoid the additional process for training a filter model. The adaptation method employs regularized optimal transport (ROT), which compensates for the error of the predicted measurement with the current measurement values to select the proper scaling parameter. In addition, the Sinkhorn–Knopp algorithm is used to minimize the cost function of ROT due to its fast convergence rate, and the convergence of the proposed ROT-based adaptive adjustment method is also analyzed. According to the analysis results of Monte Carlo simulations, it is confirmed that the proposed algorithm shows better performance than the conventional algorithms in terms of the scaling parameter selection in the UKF.

## 1. Introduction

In recent years, analysis of the state variables estimation of a dynamic system has played a central role in a variety of research areas such as navigation, target tracking, etc. [1,2,3,4,5,6,7]. Thus, filtering approaches for estimating the state variables were researched to improve the estimation performance in the case of a nonlinear system and non-Gaussian noise [8,9,10]. These methods can be classified into two categories: derivative approximations and derivative-free approximations [11]. The filtering based on derivative approximation replaces the nonlinear functions in the system description by derivative-based expansions such as the Taylor or the Fourier–Hermite series expansions [6]. The extended Kalman filter (EKF) is one of the most widely used derivative approximation methods based on first order linearization. However, the EKF can produce unstable filters in cases when local linearity is violated. Thus, derivative-free approximations based on differential polynomial interpolations, such as an unscented transform (UT) and various numerical integration rules, were recently studied for nonlinear systems [12]. The unscented Kalman filter (UKF) is a representative filtering method of derivative-free approximations that employs selected sigma-points which are propagated through a known nonlinear system model and measurement model. Some recent works on the UKF have used statistical regression instead of Taylor series linearization to increase the filter performance [13,14,15].

In the UKF, the proper positioning of the sigma points is an essential process for reducing the UT approximation error in dynamic situations. Various algorithms of the UKF have been researched on adjusting set volumes according to system dynamics with the fixed scaling parameter in the sigma point [16,17,18,19,20]. The authors proposed the adaptive UKF attempts to adaptively estimate the means and covariances of both process and measurement noises in the paper [21]. The article [22] is a moment-matching-based adaptive UKF. However, the results of recent works have indicated that the spread positions of the sigma points can be adjusted by the scaling parameter [23,24,25,26,27,28]. In the recent paper [29], the adaptation method of the scaling parameter using a random search technique for maximizing posterior probability (MAP) is applied to simultaneous localization and mapping (SLAM). Besides, adaptive UKF is applied to SLAM with adjustment of the scaling parameter for maximizing the likelihood (ML) function in the paper [30]. Similarly, the authors proposed ML as the criterion for the adaptation of the scaling parameter, and the proposed algorithm is applied to iterative multiple UKF for estimating the state of charge for lithium-ion batteries [31]. Furthermore, the adaptation of the scaling parameter using model-based optimization methods [14,15,32,33,34] leads to an improvement in the estimation performance as compared with the case in which the fixed recommended scaling parameter is used. However, the adaptation methods of the scaling parameter [27,28] may have a UT approximated error when the measurement model changes over time because the predictive measurement moments are calculated by the distribution in a fixed, pre-determined form of the measurement model. Although the model-based tuning method is performed off-line, thus incurring no extra cost for the filter during the run time, these methods require a training sequence for tuning the UT parameters, such as training a Gaussian process model.

In this paper, to more effectively deal with the change of the measurement distribution in time and avoid the additional process for training a filter model, the proposed algorithm is designed with regularized optimal transport (ROT), which compensates for the error of predicted measurement with the current measurement values. It is based on the sequential algorithm that selects the scaling parameter using the user-defined discrete set of possible values. The convergence of the proposed ROT-based adaptive adjustment method is also analyzed in this paper, and we confirm that the estimation error of the UKF with the adaptive scaling parameter remains bounded under some assumptions. ROT minimizes the cost function of transporting between a source probability distribution and the desired probability distribution using a transport map [35,36,37]. Further, the cost function is regularized by an entropic term to improve the relaxation matching between the two sample sets. Besides, the Sinkhorn–Knopp algorithm [38] is used to minimize the cost function of ROT due to its rapid convergence rate.

The rest of this paper is organized as follows. Section 2 presents a brief review of the UKF and its recommended scaling parameter. In Section 3, the adaptive scaling parameter adjustment algorithms are introduced, and the modified adaptive adjustment method using ROT is proposed. Furthermore, the stochastic stability of the proposed algorithm is analyzed. The performance analysis is carried out through Monte Carlo simulations, with the results shown in Section 4. Finally, the concluding remarks are given in Section 5.

## 2. Unscented Kalman Filter

The main algorithm of the UKF is based on the structure of the Kalman filter, where means and covariance matrices of filter state variables are recursively updated using moment approximation methods [16,27]. However, in the UKF, predicted moments are calculated by the UT. In the UT, a fixed number of sigma points that capture the desired moments (means and covariance) of the distribution of the states is deterministically selected [5]. The sigma points are then propagated with the nonlinear function, and the moments of the transformed variable are estimated [5,9]. In this section, the general filter structure of the UKF is briefly introduced.

### 2.1. Structure UT and General Filter Structure

The discrete-time nonlinear system is considered as:

(1) $$x_{k+1}=f_{k}\left(x_{k}\right)+w_{k}$$

(2) $$z_{k}=h_{k}\left(x_{k}\right)+v_{k}$$

where $x_{k}$ represents the state variable of the nonlinear system and $z_{k}$ refers to the measurement at time k, respectively. Further, $f_{k},\;\;h_{k}$ are the system and measurement model, respectively. $w_{k},\;\;v_{k}$ are the independent nonlinear system and measurement white noises, respectively, of which the probability density functions (pdfs) are assumed to be the Gaussian pdf with zero means and pre-designed covariance matrices ($Q_{k}$ and $R_{k}$, respectively). In order to estimate the desired moments of the distribution of filter states, a matrix χ of 2n+1 sigma vector $\chi_{i}$ is defined by the UT and written as follows [16]:

(3) $$\chi_{0}=\bar{x}$$

(4) $$\chi_{i}=\bar{x}+{\left(\sqrt{\left(n+\kappa \right)P_{x}}\right)}_{i},\;\;\;i=1,\;\;2,\;\;\ldots ,n$$

(5) $$\chi_{i}=\bar{x}-{\left(\sqrt{\left(n+\kappa \right)P_{x}}\right)}_{i-n},\;\;\;i=n+1,\;\;n+2,\;\;\ldots ,\;\;2n$$

where $\bar{x}$ is the mean vector of $x_{k}$, $P_{x}$ is the covariance matrix of $x_{k}$, and n is the dimension of $x_{k}$ [16]. The corresponding weights are:

(6) $$\omega_{0}=\frac{\kappa }{n+\kappa },\;\;\;\omega_{i}=\frac{\kappa }{2\left(n+\kappa \right)}$$

Sigma points $\chi_{i}$ are used in the nonlinear transformation $h_{k}$, yielding:

(7) $$\varsigma_{i}=h_{k}\left(\chi_{i}\right),\;\;\;i=0,\;\;1,\;\;2,\;\;\ldots ,2n$$

Finally, the approximated moments $z_{k}$ are expressed by:

(8) $$\begin{array}{l} {\bar{z}}_{k}\approx \sum_{i=0}^{2n}\omega_{i}\varsigma_{i},\\ P_{{\bar{z}}_{k}}\approx \sum_{i=0}^{2n}\omega_{i}\left(\varsigma_{i}-{\bar{z}}_{k}\right){\left(\varsigma_{i}-{\bar{z}}_{k}\right)}^{T}+R_{k} \end{array}$$

Using the definition of the UT, the time update and measurement update processes of the UKF are obtained [16]. According to the process of the UKF, the position of the sigma points is determined by the estimated moments and the scaling parameter, which influences the accuracy of the UT approximation [27]. Thus, the appropriate scaling parameter should be selected to improve the estimation performance.

### 2.2. Selection of Scaling Parameter

The scaling parameter, which determines the positions of the sigma points, needs to be appropriately selected to improve the estimation performance of the UKF [27,28]. In previous works, the recommended setting of the scaling parameter has been proposed as:

(9) κ=3−n

To maintain the positive semi-definition of the filter parameter κ should be selected as zero for n>3 (following the cubature Kalman filter (CKF) [8]). When the recommended scaling parameter is selected, the fourth-order term of the UT approximation error, which is only defined when the measurement model is nonlinear [27], is eliminated.

According to previous works [25,26,27,28], in some cases, the remaining terms may cause greater error than when the fourth-order term of the UT approximation error is removed. Thus, the scaling parameter can be defined as a design parameter depending on the functions in the filter state and measurement equations, characteristics of the noise, and operating point on the estimated moments.

Several studies have investigated the selection of the proper scaling parameter of the UKF, taking both an online approach [25,26,27,28] and an offline approach [23,24]. However, the offline approach for the selection of the scaling parameter causes performance degradation when it is applied to the dynamic system. Any change of the operating point in the dynamic situation requires a new training procedure for selecting the proper scaling parameter because the UT approximation error and selection of the scaling parameter depend on the estimated state statistics [27]. Thus, for selecting the scaling parameter in this paper, we focus on the online approach. In the next section, the conventional scaling parameter adjustment methods based on the online approach and the proposed scaling parameter adjustment method are introduced.

## 3. Scaling Parameter Adjustment Method

In general, the adaptive method for adjusting the scaling parameter is based on selecting the parameter that achieves the highest value of the criterion within a candidate set of scaling parameters [28]. Besides, the adaptive method is initiated between the time update process and the measurement update process of the UKF. This section briefly introduces pdf-based criteria, which use likelihood or posterior probability with predicted states′ moments [28]. However, approaches employing likelihood or posterior probability may cause a large UT approximation error when the measurement model changes over time because only the predicted values of filter states are used, with reduced emphasis on the current measurement values. Thus, a moment-based criterion based on the innovation of the filter can serve as an alternative criterion for a time-varying measurement model [28]. In the case of moment-based criterion, measurement noise has a significant effect on the criterion. Consequently, the moment-based criterion may not be suitable for adjusting the scaling parameter when the measurement noise increases. Thus, in this paper, ROT [35,36,37,38] is used to compensate for the error of predicted measurement with the current measurement values to select the appropriate scaling parameter.

### 3.1. Conventional Adjustment

The criteria for adjusting the scaling parameter used in the previous work [28] can be classified into two categories: pdf-based approach and moment-based approach. The pdf-based criteria require the pdf of the state and measurement noise [28]. The pdf of the predicted states is needed as well. Using likelihood function or posterior probability is a representative method based on the pdf-based approach. In the first method, the optimal scaling parameter is determined using the likelihood function as follows [27,28]:

(10) $$\kappa_{k}^{ML}=\underset{\kappa }{\arg\max}\;p\left(z_{k}|x_{k}\right)$$

If the predictive pdf and measurement pdf is assumed to be Gaussian, then the likelihood is obtained as:

(11) $$p\left(z_{k}|x_{k}\right)\approx N\left\{z_{k}:\;z_{k}^{-}\left(\kappa \right),P_{z,k}^{-}\left(\kappa \right)\right\}$$

where N{} refers to Gaussian distribution (normal distribution) with mean $z_{k}^{-}\left(\kappa \right)$ and covariance matrix $P_{z,k}^{-}\left(\kappa \right)$. $z_{k}^{-}\left(\kappa \right)$ is the weighted sum of the predicted measurement points which is obtained by:

(12) $$z_{k}^{-}\left(\kappa \right)=\sum_{i=0}^{2n}\omega_{i}h_{k}\left(\chi_{k,i}^{-}\right)$$

In (12), $\omega_{i}$ is the weight of the sigma points and $h_{k}\left(\chi_{k,i}^{-}\right)$ is the transformation of each sigma point, $\chi_{k,i}^{-}$, through the nonlinear function, while the predicted covariance of $h_{k}\left(\chi_{k,i}^{-}\right)$ is expressed by:

(13) $$P_{z,k}^{-}\left(\kappa \right)=\sum_{i=0}^{2n}\omega_{i}\left(h_{k}\left(\chi_{k,i}^{-}\right)-z_{k}^{-}\left(\kappa \right)\right){\left(h_{k}\left(\chi_{k,i}^{-}\right)-z_{k}^{-}\left(\kappa \right)\right)}^{T}+R_{k}$$

Equation (10) show the selected scaling parameter, which achieves the maximum value of the likelihood function within a candidate set of scaling parameters. Besides, the measurement information is only used in the criterion, and the information on the state dynamics is ignored. Thus, the criterion depends on the quality of the measurement information in a real situation. To extract the information from the known state dynamics, a posterior probability based criterion is used, presented as follows [28]:

(14) $$\kappa_{k}^{MAP}=\underset{\kappa }{\arg\max}\;p\left(x_{k}|z^{k}\right)$$

where $z^{k}=\left\{z_{1},\;z_{2},\ldots ,\;z_{k}\right\}$ and the posterior probability is decomposed into likelihood function and prior probability according to Bayes′ rule. The prior probability denotes the information of the state dynamics. If the likelihood function and prior probability are assumed to be Gaussian, then the posterior probability can be obtained as follows:

(15) $$\begin{array}{l} p\left(x_{k}|z^{k}\right)\propto p\left(z_{k}|\chi_{k}\right)p\left(\chi_{k}|z^{k-1}\right)\\ \approx N\left\{z_{k}:\;z_{k}^{-}\left(\kappa \right),P_{z,k}^{-}\left(\kappa \right)\right\}N\left\{x_{k}:\;x_{k}^{-}\left(\kappa \right),P_{x,k}^{-}\left(\kappa \right)\right\} \end{array}$$

where $x_{k}^{-}\left(\kappa \right)=\sum_{i=0}^{2n}w_{i}f_{k}\left(\chi_{k-1,i}^{+}\right)$, $f_{k}\left(\right)$ is the system model and $P_{x,k}^{-}\left(\kappa \right)=\sum_{i=0}^{2n}\omega_{i}\left(f_{k}\left(\chi_{k-1,i}^{+}\right)-x_{k}^{-}\left(\kappa \right)\right){\left(f_{k}\left(\chi_{k-1,i}^{+}\right)-x_{k}^{-}\left(\kappa \right)\right)}^{T}+Q_{k},$ $Q_{k}$ is the system noise covariance. Neither adaptive selection using likelihood nor posterior probability is suitable for selecting the correct scaling parameter when the measurement model changes over time or when the measurement is obtained using a dynamic situation because aside from the current measurement values, the adaptive selection methods only use predicted states′ moments. Thus, in previous work [28], an alternative selection of the scaling parameter using a moment-based criterion was proposed. The moment-based criterion is based on the innovation of the filter, which refers to the difference (${\bar{z}}_{k}$) between the measurement and predicted measurement and can be expressed as [28]:

(16) $$\kappa_{k}^{MNMPES}=\underset{\kappa }{\arg\min}\left[{\bar{z}}_{k}{\left(\kappa \right)}^{T}P_{{\bar{z}}_{k}}^{-1}{\bar{z}}_{k}\left(\kappa \right)\right]$$

In the case of different variances of individual measurement, the covariance of the measurement prediction error can be used to normalize the innovation square as expressed in (16). However, a moment-based criterion is sensitive to changes in measurement noise. Consequently, an incorrect scaling parameter may be selected when the measurement noise increases.

### 3.2. Regularized Optimal Transport Based Adjustment

Before explaining the proposed adaptive method, a brief review of ROT is presented. Optimal transport is proposed to minimize the cost function for transport between a source probability distribution μ and the desired probability distribution ν using a transport map [35,36,37]. In the case of discrete measures through a finite number of samples, two distributions can be written as:

(17) $$\mu =\sum_{i}p_{i}\delta_{x_{i}},\;\;\nu =\sum_{j}q_{j}\delta_{z_{j}}$$

where $\delta_{x_{i}},\;\delta_{z_{j}}$ are the Dirac at locations $x_{i},\;\;z_{j}$, respectively. Further, $p_{i},\;q_{j}$ are the probability masses associated with the i and jth sample, respectively, belonging to the condition: $\sum_{i=1}^{N_{x}}p_{i}=\sum_{j=1}^{N_{z}}q_{j}=1$. The source and desired samples are expressed as $x={\left[x_{1},\;\ldots ,\;x_{N_{x}}\right]}^{T}$ and $z={\left[z_{1},\;\ldots ,\;z_{N_{z}}\right]}^{T}$, respectively. The set of probabilistic couplings between these two distributions can then be considered as the set of doubly stochastic matrices, S defined as:

(18) $$S\left(p,\;\;q\right)=\left\{\pi \in R^{N_{x}\times N_{z}};\;\pi 1=p,\;\;\pi^{T}1=q\;\right\}$$

where 1 is an N dimensional vector of ones, and p, q are data set of the probability masses explained in (17) ($p={\left[p_{1},\;p_{2},\ldots ,p_{N_{x}}\;\right]}^{T}$, $q={\left[q_{1},\;q_{2},\ldots ,q_{N_{z}}\;\right]}^{T}$).

The Kantorovitch formulation is used to obtain the optimal transport $\pi^{*}$ as follows:

(19) $$\pi^{*}=\underset{\pi_{o}\in S}{\arg\min}\left\{\sum_{i,\;j}\pi_{ij}c_{ij}\right\}$$

where $c_{ij}$ is the cost function matrix related to the energy needed to move a probability mass from the source to the desired samples. In general, the cost is chosen as the Euclidian distance between the two samples sets as follows: $c_{ij}={\left\|x_{i}-z_{j}\right\|}^{2}$. However, optimal transport has a high computational load if the data dimension increases. In order to address the problem and improve the relaxation matching between the two sample sets, the optimal transportation problem can be regularized by an entropic term [37]. Finally, the regularized cost function can be timeously minimized using the Sinkhorn–Knopp algorithm [38], and ROT $\pi_{\gamma }^{*}$ can then be calculated as:

(20) $$\pi_{\gamma }^{*}=diag\left(v\right)Mdiag\left(u\right)$$

where $v\in R^{N_{x}\times 1},\;u\in R^{N_{z}\times 1}$ are vectors, diag(•) refers to the diagonal matrix, and $M_{ij}=\exp\left(-c_{ij}/\gamma \right)$. In addition, γ is the regularization parameter, which is the pre-designed parameter for weighting the cost. The vectors can be computed by Sinkhorn′s fixed-point iteration ($N_{r}$th iteration number) as follows:

(21) $$u_{N_{r}}=\left\{p_{i}/{\left(Mv_{N_{r}-1}\right)}_{i}\right\},\;\;\;v_{N_{r}}=\left\{q_{j}/{\left(M^{T}u_{N_{r}}\right)}_{j}\right\}$$

In this paper, the error between the $N_{z}$ of current measurements ($z_{k}^{N_{z}}$) and the predicted measurements′ points (${\tilde{z}}_{k}^{-}$) is compensated using ROT. The whole process of ROT is expressed in Table 1. The complexity of the ROT is O(NlogN) which is already analyzed in [37] (N is the dimension of the input vector).

In this process, when multiple measurements ($z_{k}^{N_{z}}$) are taken at each time sequence, the value q should be updated every epoch in order to reflect the time-varying distribution of the measurements. In this way, it is possible to easily solve by clustering a measurement dataset using Fuzzy C-means clustering [39,40] and giving a weight for setting the q based on the distance from the clustering center′s value. The weights are set using the user-defined exponential function and can be set to an appropriate value reflecting the characteristics of the system to which the algorithm is applied.

In addition, the calculation of the vectors should be performed by Sinkhorn′s fixed-point iteration to obtain the correct vectors. Using the output values of ROT, the scaling parameter is adjusted as:

(22) $$\kappa_{k}^{ROT}=\underset{\kappa }{\arg\max}\;p\left({\tilde{z}}_{k}^{+}:E\left[{\tilde{z}}_{k}^{+}\right],\;\mathrm{var}\left[{\tilde{z}}_{k}^{+}\right]\right)$$

where the expected value and covariance value of the compensated predicted measurement can be obtained as:

(23) $$E\left[{\tilde{z}}_{k}^{+}\right]=\sum_{i=0}^{2n}{\tilde{\omega }}_{k,i}{\tilde{z}}_{\;k,i}^{+}$$

(24) $$\mathrm{var}\left[{\tilde{z}}_{k}^{+}\right]=\sum_{i=0}^{2n}{\tilde{\omega }}_{k,i}\left({\tilde{z}}_{k,i}^{+}-E\left[{\tilde{z}}_{k}^{+}\right]\right){\left({\tilde{z}}_{k,i}^{+}-E\left[{\tilde{z}}_{k}^{+}\right]\right)}^{T}$$

If the measurement pdf is assumed to be Gaussian, the compensated pdf can be rewritten as:

(25) $$p\left({\tilde{z}}_{k}^{+}:E\left[{\tilde{z}}_{k}^{+}\right],\;\mathrm{var}\left[{\tilde{z}}_{k}^{+}\right]\right)\approx N\left({\tilde{z}}_{k}^{+}:E\left[{\tilde{z}}_{k}^{+}\right],\;\mathrm{var}\left[{\tilde{z}}_{k}^{+}\right]\right)$$

In general, it is not possible to identify a closed-form of an optimal solution to the criteria in (10), (14), and (22). Thus, to find a solution to the criteria, the numerical method needs to be used. While many numerical methods can be used to find the solution to the maximization of the likelihood and posterior probability, the grid method [28] is applied to identify the optimal solution to the criteria in this paper because the search area of the scaling parameter is narrow. In the grid method, the criterion used to find the solution is evaluated at the grid of the points in a feasible interval. The point with the maximal value is then selected and considered to be the recommended value of the scaling parameter. Although the simple structure of the grid method allows for its application to any algorithm, it increases the computational load on the filter. To reduce the computational costs, the adaptive selection of the scaling parameter based on the grid method is only performed when the measurement dramatically changes. The optimal scaling parameter selection of the UKF does not affect reducing the UT approximated error when the measurement model is close to the linear model.

Thus, simple logic is implemented to control the activation of adaptive adjustment logic for the scaling parameter. The logic is based on detecting whether the measurement model is close to the linear model within and around the predictive mean [28]. If such a characteristic is detected, the adaptation is skipped, and the recommended scaling parameter is used. While several methods can be used to measure the nonlinearity of the function, the simple technique based on the residual value of the weighted least squares method [28] is used in this paper to detect the nonlinearity of the measurement. The whole process of the proposed algorithm is summarized in Table 2, and its computational complexity is $O\left(N^{3}\right)($N is the dimension of the state variables).

### 3.3. Convergence of UKF with Adaptive Scaling Parameter

In this section, an analysis of the stability of the UKF with an adaptive scaling parameter is performed based on the results of previous studies [17,41]. The error of state variables and measurements are defined, respectively.

(26) $${\bar{x}}_{k}^{-}=F_{k}{\bar{x}}_{k-1}^{+}+w_{k}$$

(27) $${\bar{z}}_{k}^{}=H_{k}{\bar{x}}_{k}^{-}+v_{k}$$

where the estimation error is defined as ${\bar{x}}_{k}^{+}=x_{k}^{}-{\hat{x}}_{k}^{+}$ (after measurement update of state variable estimation), and the prediction error is written by ${\bar{x}}_{k}^{-}=x_{k}^{}-{\hat{x}}_{k}^{-}$. $F_{k}={\frac{\partial f_{k}\left(x\right)}{\partial x}|}_{x={\hat{x}}_{k}^{-}},\;\;\;H_{k}={\frac{\partial h_{k}\left(x\right)}{\partial x}|}_{x={\hat{x}}_{k}^{-}}$ are Jacobian matrices.

In the case of the UKF with an adaptive scaling parameter, if the scaling parameter changes during the process of the adjustment algorithm (that is, the location of the sigma point in the UKF changes), the prediction error of state variable and measurement are also changed simultaneously. To deal with the changes in the errors induced by the relocation of the sigma points, an instrumental diagonal matrix $\alpha_{k}=diag\left\{\alpha_{1},\;\;\alpha_{2},\cdots \;,\alpha_{n}\right\}$ for the error of state variables and $\beta_{k}=diag\left\{\beta_{1},\;\;\beta_{2},\cdots \;,\beta_{N_{z}}\right\}$ for the error of measurements are added to (26) and (27) as follows, which reflects the effect of the relocated sigma points on the predicted errors in state variables and the residual of measurements [41].

(28) $${\bar{x}}_{k}^{-}=\alpha_{k}F_{k}{\bar{x}}_{k-1}^{+}+w_{k}$$

(29) $${\bar{z}}_{k}^{}=\beta_{k}H_{k}{\bar{x}}_{k}^{-}+v_{k}$$

where $\alpha_{k}$ refers to the compensation values of the difference between true state variables and approximated state variables using the UKF with adaptive scaling parameter, particularly including state estimation error according to changes in the scaling parameter (κ). In addition, above, (28) and (29) are valid only when there are additional errors in the filter models, respectively.

In (26), the predicted error is defined, and it is written in detail as follows:

(30) $$\begin{array}{l} {\bar{x}}_{k}^{-}=x_{k}-{\hat{x}}_{k}^{-}\\ \;\;\;\;\;\;=x_{k}-{\sum_{i=0}^{2n}\omega_{i}f_{k}\left(\chi_{i}\right)|}_{{\bar{x}}_{k}={\hat{x}}_{k-1}^{+}}\\ \;\;\;\;\;\;=x_{k}-\omega_{0}f_{k}\left({\hat{x}}_{k-1}^{+}\right)-\sum_{i=1}^{n}\omega_{i}f_{k}\left({\hat{x}}_{k}^{+}+{\left(\sqrt{\left(n+\kappa \right)P_{x}}\right)}_{i}\right)\\ \;\;\;\;\;\;\;\;\;\;-\sum_{i=1}^{2n}\omega_{i}f_{k}\left({\hat{x}}_{k-1}^{+}-{\left(\sqrt{\left(n+\kappa \right)P_{x}}\right)}_{i-n}\right) \end{array}$$

Let ${\left(\sqrt{\left(n+\kappa \right)P_{x}}\right)}_{i}=\Delta x_{i}$ and take the Taylor series expansion of the nonlinear transformation of f(x), then the above equation can be derived as:

(31) $$\begin{array}{l} {\bar{x}}_{k}^{-}=x_{k}-f_{k}\left({\hat{x}}_{k-1}^{+}\right)+\frac{n}{n+\kappa }f_{k}\left({\hat{x}}_{k-1}^{+}\right)\\ \;\;\;\;\;\;\;\;\;\;-\sum_{i=1}^{n}\frac{n}{2\left(n+\kappa \right)}f_{k}\left({\hat{x}}_{k-1}^{+}+\Delta x_{i}\right)\\ \;\;\;\;\;\;\;\;\;\;-\sum_{i=1}^{n}\frac{1}{2\left(n+\kappa \right)}f_{k}\left({\hat{x}}_{k-1}^{+}-\Delta x_{i}\right)\;\; \end{array}$$

(32) $$\;\;\;\;=x_{k}-f_{k}\left({\hat{x}}_{k-1}^{+}\right)-\sum_{i=1}^{n}\frac{1}{n+\kappa }\left\{\frac{D_{\Delta x_{i}}^{2}}{2!}f_{k}+\frac{D_{\Delta x_{i}}^{4}}{4!}f_{k}+\cdots \right\}\;$$

In (31), the Taylor series expansion of $f_{k}\left({\hat{x}}_{k-1}^{+}+\Delta x_{i}\right)\;=f_{k}\left({\hat{x}}_{k-1}^{+}\right)+D_{\Delta x_{i}}^{}f_{k}+\frac{D_{\Delta x_{i}}^{2}}{2!}f_{k}+\frac{D_{\Delta x_{i}}^{4}}{4!}f_{k}+\cdots \;$ and $D_{\Delta x_{i}}^{m}f_{k}={{\left(\Delta x_{i}•\nabla_{x}\right)}^{m}f_{k}\left(x\right)|}_{{\bar{x}}_{k}={\hat{x}}_{k-1}^{+}}={\left\{\sum_{j=1}^{N_{x}}\Delta x_{i}•\frac{\partial }{\partial x_{j}}\right\}}^{m}{f_{k}\left(x\right)|}_{{\bar{x}}_{k}={\hat{x}}_{k-1}^{+}}$.

Finally, in (32), $x_{k}-f_{k}\left({\hat{x}}_{k-1}^{+}\right)\approx F_{k}{\bar{x}}_{k-1}^{+}$ and the rest of the terms are modeled by a function of the scaling parameter.

(33) $${\bar{f}}_{k}\left(\kappa \right)=\sum_{i=1}^{n}\frac{1}{n+\kappa }\left\{\frac{D_{\Delta x_{i}}^{2}}{2!}f_{k}+\frac{D_{\Delta x_{i}}^{4}}{4!}f_{k}+\cdots \right\}$$

Then, the predicted error has the relationship between $\alpha_{k}$ and scaling parameter κ, as follows:

(34) $${\bar{x}}_{k}^{-}\approx F_{k}{\bar{x}}_{k-1}^{+}-{\bar{f}}_{k}\left(\kappa \right)=\alpha_{k}F_{k}{\bar{x}}_{k-1}^{+}+w_{k}$$

(35) $$\alpha_{k}=I-\left({\bar{f}}_{k}\left(\kappa \right)+w_{k}\right){\left(F_{k}{\bar{x}}_{k-1}^{+}\right)}^{T}{\left(F_{k}{\bar{x}}_{k-1}^{+}{\bar{x}}_{k-1}^{+}{}^{T}F_{k}^{T}\right)}^{-1}$$

where I is the $N_{x}\times N_{x}$ identity matrix. Likewise, the relationship between $\beta_{k}$ and κ can be obtained like the above process (from (27) to (35)). For convenient analysis of the stability of the UKF with the adaptive scaling parameter, approaches used in previous works [17,41] are employed to simplify the error expression. In addition, some assumptions are held for verifying the boundedness of the estimation errors in the UKF.

There exist real constants which are related to the system model and measurement model written in (1) and (2):

(36) $$f_{\min,\;\;}f_{\max,\;\;}h_{\min,\;\;}h_{\max,\;\;}q_{\min,\;\;}q_{\max,\;\;}p_{\min,\;\;}p_{\max,\;\;}R_{\max\;\;}>0$$

(37) $$\alpha_{\min,\;\;}\alpha_{\max,\;\;}\beta_{\min,\;\;}\beta_{\max,\;\;}T_{\kappa }>0$$

such that the following bounds on matrices of filter models are satisfied for every time index, k as follows:

(38) $$f_{\min}^{2}I\leq F_{k}F_{k}^{T},\;\;\;h_{\min}^{2}I\leq H_{k}H_{k}^{T},\;\;\;\;\left\|F_{k}\right\|\leq f_{\max\;\;}$$

(39) $$\left\|H_{k}\right\|\leq h,\;\;\;q_{\min}I\leq Q_{k}^{*},\;\;\;R_{k}\leq r_{\max}I$$

where ‖‖ is matrix norm.

According to (33), the value ${\bar{f}}_{k}\left(\kappa \right)$ is related to the power series of n+κ and error covariance, $P_{x}$ as well as the partial differential of the system model, $D_{\Delta x_{i}}^{m}$, using the predicted state variable, ${\hat{x}}_{k-1}^{+}$, at each time step. Among these values, the influence of the scale parameter, κ on the amount of ${\bar{f}}_{k}\left(\kappa \right)$ increases with the order of the power series (m). Thus, the boundary of the ${\bar{f}}_{k}\left(\kappa \right)$′s value is approximately set to $\left\|{\bar{f}}_{k}\left(\kappa \right)\right\|\leq T_{\kappa }$, and finally, the boundary of $\alpha_{k}$ and $\beta_{k}$ is:

(40) $$\left\|\alpha_{k}\right\|\leq \sqrt{n}\left|1-\frac{\left(T_{\kappa }+\sqrt{q_{\max}}\right)\sqrt{P_{\max}}}{f_{\max}P_{\max}}\right|=\alpha_{\max},\;\;\;\left\|\beta_{k}\right\|\leq \beta_{\max}$$

The relationship between $T_{\kappa }$ and κ, while it will depend on the system model and the estimated value, is generally proportional.

The predicted error covariance matrix of state variables, $P_{k}^{-}$, is defined as follows:

(41) $${\hat{P}}_{k}^{-}=\left[\alpha_{k}F_{k}\left(I-K_{k}\beta_{k}H_{k}\right)\right]{\hat{P}}_{k-1}^{-}{\left[\alpha_{k}F_{k}\left(I-K_{k}\beta_{k}H_{k}\right)\right]}^{T}+Q_{k}^{*}$$

where $Q_{k}^{*}=Q_{k}^{}+\alpha_{k}F_{k}K_{k}R_{k}{\left(\alpha_{k}F_{k}K_{k}\right)}^{T}+\delta P_{k}$ and $\delta P_{k}$ refer to the error between the ideal error covariance matrix and the unscented transformed error covariance matrix [41].

The weighted error square of state variables is defined as:

(42) $$e_{k}\left({\bar{x}}_{k}^{-}\right)={\left({\bar{x}}_{k}^{-}\right)}^{T}{\left({\hat{P}}_{k}^{-}\right)}^{-1}{\bar{x}}_{k}^{-}$$

Using the above two equations, the expectation of the weighted error square is obtained by:

(43) $$\begin{array}{l} E\left\{e_{k}\left({\bar{x}}_{k}^{-}\right)|{\bar{x}}_{k-1}^{-}\right\}={\left({\bar{x}}_{k}^{-}\right)}^{T}{\left[\alpha_{k}F_{k}\left(I-K_{k}\beta_{k}H_{k}\right)\right]}^{T}{\left({\hat{P}}_{k}^{-}\right)}^{-1}\\ \;\;\;\;\;\;\;\;\;\;\;\;\;\;\;\;\;\;\;\;\;\;\;\;\;\;\;\;\;\;\;\;\;\;\;\;\;\;\;\times \left[\alpha_{k}F_{k}\left(I-K_{k}\beta_{k}H_{k}\right)\right]{\bar{x}}_{k}^{-}\\ \;\;\;\;\;\;\;\;\;\;\;\;\;\;\;\;\;\;\;\;\;\;\;\;\;\;\;\;\;\;\;\;\;\;\;\;\;\;\;+E\left\{v_{k}^{T}{\left[\alpha_{k}F_{k}K_{k}\right]}^{T}{\left({\hat{P}}_{k}^{-}\right)}^{-1}\left[\alpha_{k}F_{k}K_{k}\right]v_{k}^{}|{\bar{x}}_{k-1}^{-}\right\}\\ \;\;\;\;\;\;\;\;\;\;\;\;\;\;\;\;\;\;\;\;\;\;\;\;\;\;\;\;\;\;\;\;\;\;\;\;\;\;\;+E\left\{w_{k}^{T}{\left({\hat{P}}_{k}^{-}\right)}^{-1}w_{k}^{}|{\bar{x}}_{k-1}^{-}\right\} \end{array}$$

According to the results of previous works [17,41], the expectation of the weighted error square is bounded as:

(44) $$\begin{array}{l} E\left\{e_{k}\left({\bar{x}}_{k}^{-}\right)|{\bar{x}}_{k-1}^{-}\right\}\leq \\ {\left[1+\frac{q_{\min}I}{{\left(a_{\max}f_{\max}+a_{\max}f_{\max}K_{\max}\beta_{\max}h_{\max}\right)}^{2}-p_{\max}}\right]}^{-1}e_{k}\left({\bar{x}}_{k}^{-}\right)\\ +\frac{K_{\max}^{2}a_{\max}^{2}f_{\max}^{2}q_{\max}^{}N_{x}}{p_{\min}}+\frac{r_{\max}^{}N_{z}}{p_{\min}} \end{array}$$

If the coefficient of $e_{k}\left({\bar{x}}_{k}^{-}\right)$ is replaced with 1−λ and the last term is substituted with an arbitrary positive value μ, (42) can be rewritten as:

(45) $$\begin{array}{l} E\left\{e_{k}\left({\bar{x}}_{k}^{-}\right)|{\bar{x}}_{k-1}^{-}\right\}\leq \left(1-\lambda \right)e_{k}\left({\bar{x}}_{k}^{-}\right)+\mu \\ E\left\{e_{k}\left({\bar{x}}_{k}^{-}\right)|{\bar{x}}_{k-1}^{-}\right\}-e_{k}\left({\bar{x}}_{k}^{-}\right)\leq -\lambda e_{k}\left({\bar{x}}_{k}^{-}\right)+\mu \end{array}$$

Finally, applying Lemma 2.1 of the previous work [42] to (49), the stochastic process of ${\bar{x}}_{k}^{-}$ is bounded. Therefore, it is shown that the estimation error of the UKF with the adaptive scaling parameter remains bounded if the filter system satisfies some assumptions listed in (36) to (40).

## 4. Simulation

The problem of bearings-only tracking arises in a variety of important practical applications, and it is often used for evaluating the performance of nonlinear filters [43]. The simple 2-D example describing the bearings-only tracking [27] is used to compare the performance of the proposed UKF algorithm with that of the CKF [8], the iterated Kalman filter (IKF) based on the UT [13,15], which has a method similar to that of the proposed algorithm with linearization considered in the update step. In addition, the UKF-AA algorithm [28,29,30,31], using the conventional scaling adjustment method mentioned in Section 3.1, is also used to compare the performance of the proposed algorithm. The system and measurement model are defined as:

(46) $$x_{k+1}=\left[\begin{array}{cc} 0.9 & 0\\ 0 & 1 \end{array}\right]x_{k}+w_{k}$$

(47) $$z_{k}^{i}=\left\{z_{k}^{1},z_{k}^{2},\ldots ,z_{k}^{i}\right\}$$

(48) $$p\left(z_{k}^{i}\right)=N\left(\tan^{-1}\left(\frac{x_{2,k}-\sin\left(k\right)}{x_{1,k}-\cos\left(k\right)}\right),R_{k}\;\;\right)$$

where the filter state variables are the 2-D position $x_{k}={\left[x_{1,k},\;x_{2,k}\right]}^{T}$, $k=1,\;\;\ldots ,N_{s}$, $N_{s}=500$, $Q_{k}=E\left[w_{k}w_{k}^{T}\right]$, $Q_{k}=\left[\begin{array}{cc} 0.1 & 0.05\\ 0.05 & 0.1 \end{array}\right]$, $R_{k}=0.025$, ∀k, and i is the number of measurements of which the distribution $p\left(z_{k}^{i}\right)$ is assumed to be Gaussian with a mean of $\tan^{-1}\left(\frac{x_{2,k}-\sin\left(k\right)}{x_{1,k}-\cos\left(k\right)}\right)$ and variance of $R_{k}$. The initial values of the filter are set as follows: $x_{0}={\left[20,\;\;5\right]}^{T}$, $P_{0}=\left[\begin{array}{cc} 0.1 & 0\\ 0 & 0.1 \end{array}\right]$, i=300. The candidate scaling parameter set is assumed to be {0,1,2,3,4}. The performance of the filter is analyzed in terms of mean squared error (MSE) using Monte Carlo simulations with 500 independent runs. Further, the averaged normalized estimation error squared (ANEES) is also used to analyze the performance of the proposed algorithm and is defined as:

(49) $$ANEES_{k}=\frac{1}{N_{s}}\sum_{j=1}^{N_{s}}\left({\left(x_{k}^{j}-{\hat{x}}_{k}^{j}\right)}^{T}{\left({\left(P_{k}^{+}\right)}^{j}\right)}^{-1}\left(x_{k}^{j}-{\hat{x}}_{k}^{j}\right)\right)$$

ANEES provides an evaluation of a relative estimation error at the j-th Monte Carlo simulations ($N_{s}$ is the number of total runs and is set to 500), and it evaluates a self-assessment provided by each filter in the form of the covariance matrix of the estimation error.

**Figure 1:**
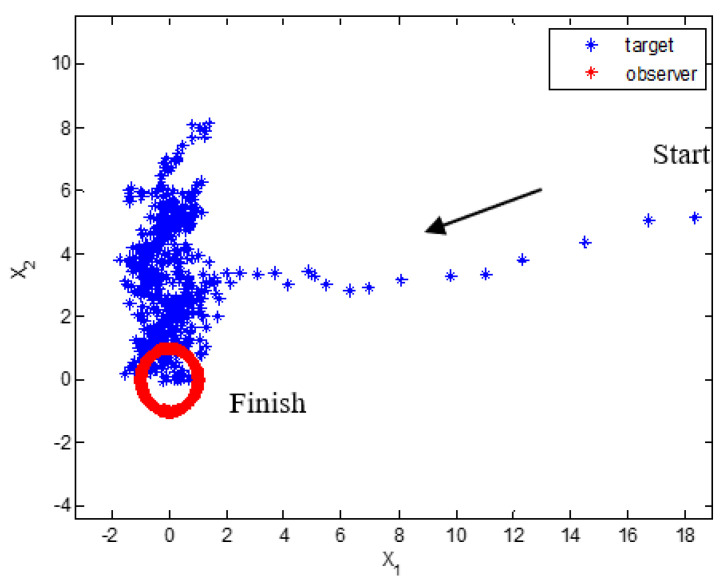
Target and observer trajectory.

**Figure 2:**
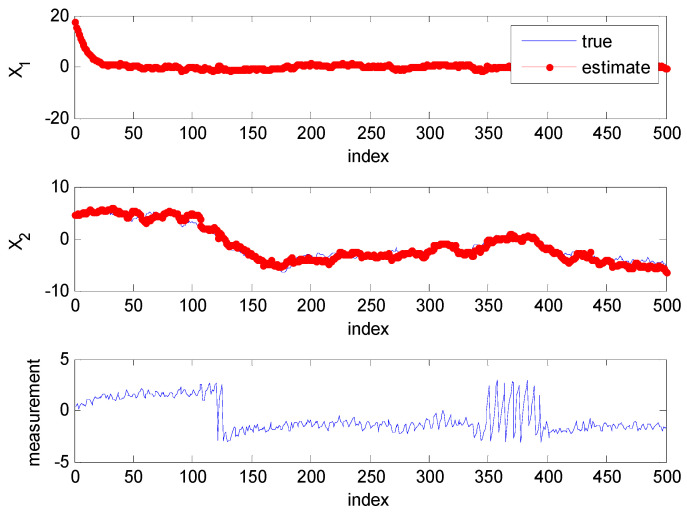
Estimate results measurement (**top**: the first state estimate results, **middle**: the second state estimate results, **bottom**: the expected value of measurement).

**Figure 3:**
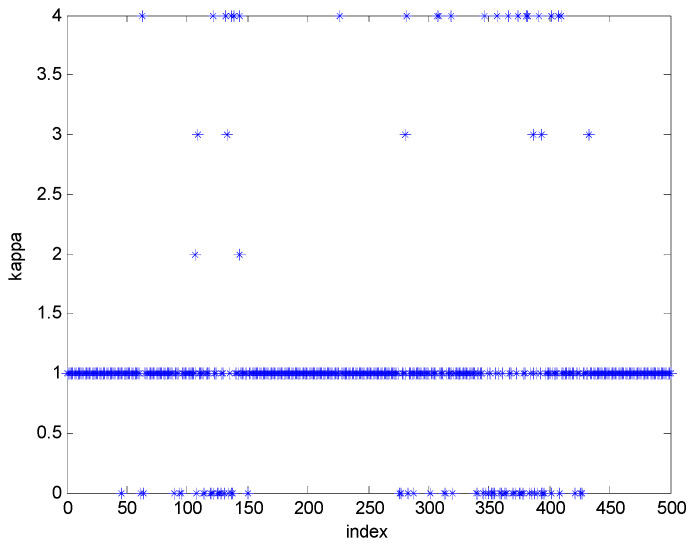
Scaling parameter adjustment results (minimum value of the scaling parameter: 0, maximum value of the scaling parameter: 4).

Figure 1 show one target and observer trajectory of the Monte Carlo simulation. The target moves from the right side to the left side during the first time and holds a static position with a randomly varying vertical position during the remaining simulation time. The trajectory of the target is plotted with asterisks, while the path of the observer is plotted with dots. The dynamic model of the target, which is sensitive to changes in the scaling parameter of the UKF, is based on the original paper of UKF-AA [28]. Besides, the circular trajectory of the observer is designed to give a significant change in the measurement values. The information set of direction between target and observer is obtained according to the change of the observer′s position. Figure 2 refer to the estimation results of the two-state variables and the expected value of measurement. These results are obtained in the simulation case, as shown in Figure 1. The proposed algorithm accurately tracks the target, although the measurement changes in the 0~50 (related to $x_{1}$) and the 50–450 time indexes (especially 100~150, 250~450 time related to $x_{2}$). During these periods, the scaling parameter adjustment logic is activated to reduce the UT approximated error, as shown in Figure 3. In other periods, the detection algorithm of measurement nonlinearity suggests that the behavior of the measurement is linear. Consequently, the scaling parameter is set to the recommended scaling parameter, which is 1 in the case of two-dimensional states.

**Figure 4:**
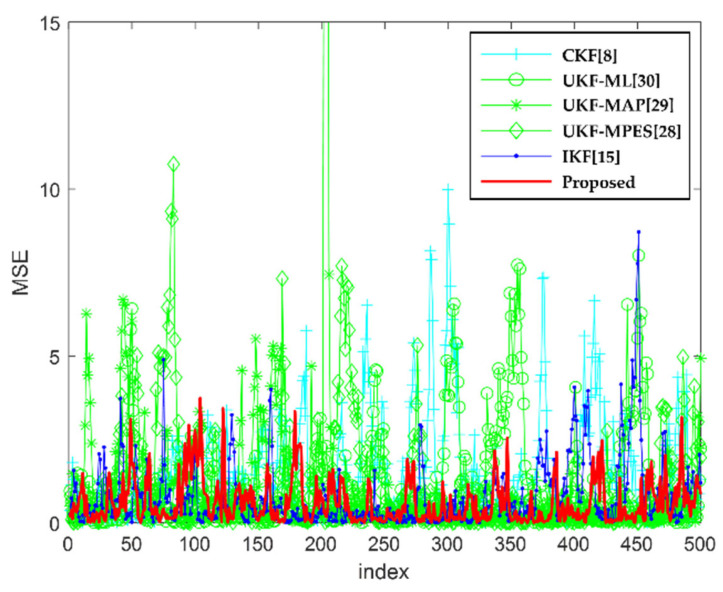
The MSE of each filter.

**Figure 5:**
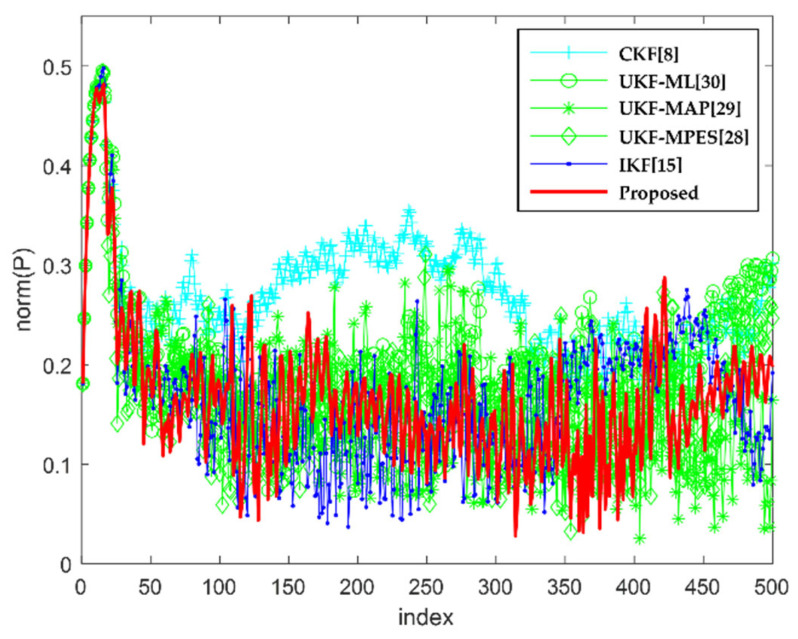
The matrix norm of error covariance matrix according to each filter.

Figure 4 and Figure 5 show the result of the MSE and the matrix norm of the error covariance matrix, respectively. These figures are to facilitate the assessment of estimation consistency. For verifying the performance, the proposed method is compared with various existing methods. In the CKF [8], the scaling parameter is always set to zero. In the UKF-AA [28], the conventional scaling parameter adjustment method is used in three types, which are the maximization of likelihood (ML) [30,31], posterior probability (MAP) [29], and predicted error square (MPES). Besides, the IKF is based on the UT [13,15], with linearization considered in the update step. Although the primary concept of this algorithm was implemented for performance analysis, the detailed settings for an application to this system differed from the original setting in the previous work [15]. The results of these figures show that the proposed method has the smallest estimation error and that all the filters maintained the estimate consistency.

Table 3 show the MSE and ANEES of the Monte Carlo simulation for efficient performance comparison. In Table 3, ROT refers to the ROT based proposed method, and SP refers to the scaling parameter of the UKF. It is confirmed that the proposed algorithm shows better performance compared with CKF, UKF-AA, and IKF because the proposed scaling parameter selection algorithm based on ROT uses the predicted values of filter states with the current measurement values so that the UT approximated error is reduced when the measurement values change rapidly.

Considering that the proposed algorithm can be applied to other systems, relative operating time and related parameters were summarized in Table 4 using a function provided by the simulation program (MATLAB function, tic/toc used). In Table 4, total operating-time refers to the time taken to process a sequence of a simulation, and scaling parameter adjustment-time means the time it takes to adjust parameters during a sequence of a simulation. The rate indicates the ratio of the scaling parameter adjustment-time to total operating-time.

As a result of comparing the algorithm processing time, the parameter adjustment step of the proposed method has an extended processing time. Thus, it is necessary to improve the processing speed of the algorithm to apply it to a real system even if the proposed method has the best estimation performance.

## 5. Conclusions

The UKF with a novel scaling parameter adaptation method using ROT is proposed to improve the estimation performance of the UKF when the measurement changes rapidly. In addition, the Sinkhorn–Knopp algorithm is used to minimize the cost function of ROT due to its fast convergence rate and the relaxation matching between predicted measurement sets and received measurement sets. Monte Carlo simulations are performed to evaluate the estimation performance of the proposed algorithm, and the results confirm that the proposed algorithm performs better than the UKF with conventional scaling parameter adaption methods. However, in order to apply the proposed algorithm to the actual tracking system, it is necessary to study additional algorithms that can reduce the operation time of the scaling parameter adaptation method, which occupies 69% of the operation time.

## Figures and Tables

**Table 1:** Pseudocode of the regularized optimal transport.

**Input:**	$\mathrm{Measurements}:\;z_{k}^{N_{z}}=\left\{z_{k}^{1},\;z_{k}^{2},\;\;\ldots \;\;,\;z_{k}^{N_{z}}\right\}$
	$\mathrm{Predicted}\;\mathrm{measurement}\;\mathrm{points}\;\mathrm{set}\;({\tilde{z}}_{k}^{-}=h_{k}\left(\chi_{k}^{-})\right)$
	$\mathrm{Sigma}\;\mathrm{points}\prime \;\mathrm{weight}\;\mathrm{vector}\;(\omega_{k})$ using (6)
	Regularization parameter (γ)
**Output:**	$\mathrm{Compensated}\;\mathrm{point}\;\mathrm{set}\;\mathrm{of}\;\mathrm{predicted}\;\mathrm{measurement},\;{\tilde{z}}_{k}^{+}$
	$\mathrm{Revalued}\;\mathrm{weights},\;{\tilde{\omega }}_{k}$
**Initialization**	
1	$v_{0}=1,\;\;v_{0}\in R^{N_{x}}$
Marginal values calculation	
2	$p=N_{z}\omega_{k},\;\;q=1,\;\;q\in R^{N_{z}}$
Clustering and updating q in the measurement update	
M calculation	
3	$M_{ij}=e^{-\gamma {\left\|z_{k}^{i}-{\tilde{z}}_{\;k,j}^{-}\right\|}^{2}}$ $,\;M\in R^{N_{z}\times N_{x}}$
$\mathrm{Vectors}\;\mathrm{calculation}\;\mathrm{using}\;(21):\;u_{k}=u_{N_{r}},\;\;\;v_{k}=v_{N_{r}}$	
Optimal map calculation	
4	$\pi_{k}=diag\left(v_{k}\right)Mdiag\left(u_{k}\right)$
5	${\tilde{z}}_{k}^{+}={\tilde{z}}_{k}^{-}\pi_{k}$
6	${\tilde{\omega }}_{k}=\frac{1}{N_{z}}\pi_{k}1$

**Table 2:** Pseudocode of the proposed algorithm.

**Input:**	$\left[x_{k-1}^{+},P_{k-1}^{+}\right]$ (k−1 step estimates)
**Output:**	$\left[x_{k}^{+},P_{k}^{+}\right]$ (k step estimates)
**UKF prediction:**	$\left[x_{k}^{-},P_{k}^{-},z_{k}^{-},\;S_{k}^{-}\right]=\mathrm{UKF}\_\mathrm{prediction}\left(x_{k-1}^{+},P_{k-1}^{+}\right)$
1	Sigma points calculation
2	${\left\{\chi_{k-1}^{+}\right\}}_{0}=x_{k-1}^{+}$
3	${\left\{\chi_{k-1}^{+}\right\}}_{j}=x_{k-1}^{+}+{\left(\sqrt{\left(n+\kappa \right)P_{x}{}_{k-1}^{+}}\right)}_{j},\;\;\;j=1,\;\;2,\;\;\ldots ,n$
4	${\left\{\chi_{k-1}^{+}\right\}}_{j}=x_{k-1}^{+}-{\left(\sqrt{\left(n+\kappa \right)P_{x}{}_{k-1}^{+}}\right)}_{j-n},\;\;j=n+1,\;\ldots ,\;\;2n$
5	Weight calculation
6	$\omega_{0}=\frac{\kappa }{n+\kappa },\;\;\;\omega_{j}=\frac{\kappa }{2\left(n+\kappa \right)}$
7	Prediction values calculation
8	$\chi_{k}^{-}=f\left(\chi_{k-1}^{+}\right)$
9	$x_{k}^{-}=\sum_{j=0}^{2n}\omega_{j}{\left\{\chi_{k}^{-}\right\}}_{j}$ $,\;P_{k}^{-}=\sum_{j=0}^{2n}\omega_{j}\left[{\left\{\chi_{k}^{-}\right\}}_{j}-x_{k}^{-}\right]{\left[{\left\{\chi_{k}^{-}\right\}}_{j}-x_{k}^{-}\right]}^{T}$
Scaling parameter adjustment:	
10	${\tilde{z}}_{k}^{+}=ROT\left(z_{k}^{N_{z}},{\tilde{z}}_{k}^{-},\;\omega_{k},\;\gamma \;\right)$ written in Table 1
11	$\kappa_{k}^{ROT}=\underset{\kappa }{\arg\max}\;p\left({\tilde{z}}_{k}^{+}:E\left[{\tilde{z}}_{k}^{+}\right],\;\mathrm{var}\left[{\tilde{z}}_{k}^{+}\right]\right)$ in (23)
$\mathrm{UKF}\;\mathrm{update}:\;\left[x_{k}^{+},P_{k}^{+}\right]=\mathrm{UKF}\_\mathrm{update}\left(x_{k}^{-},{\hat{z}}_{k}^{+}\right)$	
12	$\mathrm{Sigma}\;\mathrm{points}\;\mathrm{recalculation}\;\mathrm{with}\;\kappa_{k}^{ROT}$ (the process in 1~4)
13	$\mathrm{Weight}\;\mathrm{recalculation}\;\mathrm{with}\;\kappa_{k}^{ROT}$ (the process in 6)
14	$\mathrm{Prediction}\;\mathrm{values}\;\mathrm{recalculation}\;\mathrm{with}\;\kappa_{k}^{ROT}$ (the process in 8~9)
15	Measurement prediction
16	$z_{k}^{-}=\sum_{j=0}^{2n}\omega_{j}{\left\{{\tilde{z}}_{k}^{-}\right\}}_{j}$ $,\;S_{k}^{-}=\sum_{j=0}^{2n}\omega_{j}\left[{\left\{{\tilde{z}}_{k}^{-}\right\}}_{j}-z_{k}^{-}\right]{\left[{\left\{{\tilde{z}}_{k}^{-}\right\}}_{j}-z_{k}^{-}\right]}^{T}$
17	$P_{x,y}{}_{\;k}^{}=\sum_{i=0}^{2n}\omega_{i}\left[{\left\{\chi_{k}^{-}\right\}}_{i}-x_{k}^{-}\right]{\left[{\left\{{\tilde{z}}_{k}^{-}\right\}}_{i}-z_{k}^{-}\right]}^{T}$
18	Update values calculation
19	$K_{k}=P_{x,y}{}_{\;k}^{}{\left(S_{k}^{-}\right)}^{-1}$
20	$x_{k}^{+}=x_{k}^{-}+K_{k}\left(z_{k}-z_{k}^{-}\right)$ $,\;P_{k}^{+}=P_{k}^{-}-K_{k}S_{k}^{-}K_{k}^{T}$

**Table 3:** Comparison results of the filter performance.

Types	CKF	UKF-AA			IKF	Proposed
SP	0	ML	MAP	MPES		ROT
MISE	5.973	5.400	5.370	5.901	4.079	3.876
ANEES	7.188	6.795	5.571	7.102	4.213	4.193

**Table 4:** Operating-time of the filter performance.

Types	UKF-AA			Proposed
SP	ML	MAP	MPES	ROT
Total operating-time (s)	0.0364	0.0381	0.0358	0.0406
Scaling parameter adjustment-time (s)	0.0208	0.0257	0.0193.	0.0280
Rate (%)	57.1114	67.5307	53.9106	68.9655

## Data Availability

Not applicable.
